# Sellar B lymphoblastic lymphoma mimics pituitary apoplexy with newly discovered gene mutations in TP53 and PAX5: A case report

**DOI:** 10.3389/fonc.2023.1087232

**Published:** 2023-02-07

**Authors:** Yi Wang, Yajun Yang, Qiuxia Wang, Ce Wang, Xinjuan Xu, Dongao Wang, Feirong Bai, Panpan Li, Xintao Huang

**Affiliations:** ^1^ Department of Neurosurgery, First Hospital of Shanxi Medical University; and The First School of Clinical Medicine, Shanxi Medical University, Taiyuan, Shanxi, China; ^2^ Department of Gastroenterology, Affiliated Hospital of Southwest Medical University, Luzhou, Sichuan, China; ^3^ Department of Neurosurgery, Shanxi Cardiovascular Disease Hospital, Taiyuan, Shanxi, China; ^4^ Department of Neurosurgery, First Hospital of Shanxi Medical University, Taiyuan, Shanxi, China

**Keywords:** B-LBL, sellar tumor, rare, gene mutation, Pax5, TP53

## Abstract

Lymphoblastic lymphoma (LBL) is a rare tumor that accounts for approximately 2-4% of all non-Hodgkin lymphomas, and less than 20% of LBLs are derived from B cells. B- Lymphoblastic lymphoma (B-LBL) often presents as bone marrow and peripheral blood lesions, and is very rare to present as a seller mass. We report a case of sellar B lymphoblastic lymphoma mimicking pituitary apoplexy, and review its diagnosis and treatment process, combined with the literature to deepen the understanding of sellar tumors.

## Introduction

Lymphoblastic lymphoma (LBL) is a rare type of aggressive non‐Hodgkins lymphoma (NHL). In the 2017 WHO classification, LBL is classified together with acute lymphoblastic leukemia (ALL) as ALL/LBL, and LBL is conventionally distinguished from ALL by less than 20–25% marrow infltrating blasts cells (1). LBL represents 2-4% of adult NHL, divided into B cell LBL (B-LBL) and T cell LBL (T-LBL), but only 20% of them are derived from precursor B cells (2). The global incidence of B-LBL/ALL is about 1-5/100000 persons per year (3). Up to now, only one case of B-LBL in sellar has been reported and diagnosed as primary pituitary stalk B-LBL (4). Due to the extreme rarity of sellar B-LBL, its incidence and prevalence cannot be estimated.

## Case presentation

**Figure 1:**
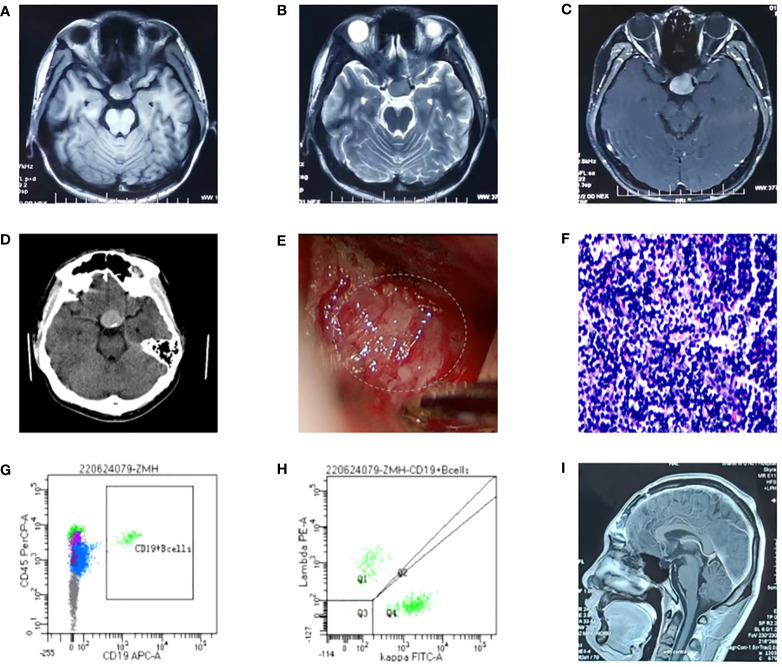
**(A-C)** Head MRI: the sella was expanded, with an oval slightly long T1 and long T2 abnormal signal, and there was uneven enhancement and a clear boundary. The neurohypophysis is clearly shown, the left optic chiasm is compressed, and the internal carotid artery in the left cavernous sinus is partially surrounded. The intracranial segment of the left optic nerve were compressed and slightly thinned, and the enhanced scan showed mild enhancement; **(D)** Head CT: the sella is enlarged, high-density nodules are seen in the pituitary fossa, the size is about 2.1*2.8cm, and the CT value is about 56HU; **(E)** The tumor was found to have abundant blood supply during the operation; **(F)** Pathological section: Proliferating tumor cells and have a starry sky appearance and they are dense, with medium cell volume, thin cytoplasm, large and slightly irregular nuclei, and fine chromatin; **(G, H)** Flow cytometry, and we found that B lymphocytes with CD19+CD20+ accounted for about 19.8% of lymphocytes, with polyclonal expression of Kappa and Lambda; **(I)** Postoperative re-examination of head MRI showed no tumor recurrence.

A 22-year-old male was presented to our hospital in May 2022 with “sudden drop of left eye vision for one week.” The patient had been healthy prior to the onset of his illness, and denied family history of genetic disease. Visual assessment: visual acuity was 0.2 in the left eye and 1.0 in the right eye. Fundus examinations disclosed optic disc edema in the left eye. Acuity examinations showed inferonasal sector hemianopia in the left eye and superonasal sector hemianopia in the right eye. He underwent MRI at another hospital 2 days before admission; Results showed that the sella was expanded, with an oval slightly long T1 and long T2 abnormal signal, and there was uneven enhancement and a clear boundary; The lesion size was 2.16x1.60x2.16cm (**Figures 1A–C**). A diagnosis of a large pituitary macroadenoma with compression of the left optic chiasm was considered. His disease progressed rapidly. On the afternoon of admission, he presented with a sudden decrease in visual acuity in his right eye, and his visual acuity of the right eye improved slightly after rapid infusion of mannitol and dexamethasone. CT of the head showed that a hyperdense nodules can be seen inside the pituitary fossa (**Figure 1D**). We considered it to be a pituitary apoplexy. Then, an emergency craniotomy was performed. Preoperative tests: 1. Blood routine examination: leucocyte [8.6×10^9^/L (3.5-9.5)], absolute value of lymphocytes [1.22×10^9^/L (1.1-3.2)], lymphocyte percentage [14.3% (20-50)]; 2. endocrine tests: cortisol [1174nmol/L (171-536)] was elevated, and other hormones were normal; 3. HIV (-). We found the tumor had an outer envelope, with tough texture and a very rich blood supply during surgery (**Figure 1E**). All eyes had significantly improved vision without diabetes insipidus and electrolyte disturbance after surgery. Pathological results: B-lymphoblastic lymphoma (**Figure 1F**). Immunohistochemistry results: CD10(+ +), CD79A (+), KI67(+ 90%), P53(10% +), TDT (65%), Syn (+), BCL-6(+), BCL-2(> 90% +), LMO2(+), PAX5(+), CD99(+), CD20(-). Bone marrow specimens were evaluated by flow cytometry, and we found that B lymphocytes with CD19+CD20+ accounted for about 19.8% of lymphocytes, with polyclonal expression of Kappa and Lambda (**Figures 1G, H**), but no obvious abnormal immunophenotype cells were found. Next-generation sequencing was performed on tumor tissue to detect of 93 gene variants associated with lymphoma, and six variations in five genes were found (**Table 1**). *PAX5* c.913_923delCGTGACTTGGC (p.R305Efs*32) and *TP53* c.969_971delGGAinsAGGC (p.D324Gfs*13) are not recorded in COSMIC, MSK database, and both of them are new discoveries. One month after surgery, he underwent a PET/CT examination, which showed that the tumor had invaded both kidneys, ureters, prostate, pelvis, and left femoral head and neck. There was no tumor recurrence when he had his second MRI in October 2022 (**Figure 1I**). On October 26, 2022, the patient had a re-examination of PET/CT and found that the increased FDG uptake in his both kidneys, ureters, prostate, pelvis, and left femoral head and neck were reduced compared with the previous range, and the extent of lymphoma involvement was relieved. Until mid-December, he had been receiving 4 rounds of hyper-CVAD chemotherapy at our institution, and complications such as acute pancreatitis and anemia occurred. The patient now claims that his left eye vision is worse than postoperative and cannot take care of himself. Next step he is going to undergo stem cell transplant treatment.

**Table 1:** Genome variation detection results.

Gene	Transcript	Base changes	Amino acid changes	Functional regions	Variation frequency/copy number
*PAX5*	NM_016734.1	c.77T>G	p.V26G	EX2	45.8%
*PAX5*	NM_016734.1	c.913_923del CGTGACTTGGC	p.R305Efs*32	EX8	33.8%
*TP53*	NM_000546.5	c.969_971del GGAinsAGGC	p.D324Gfs*13	EX9	3.8%
*XPO1*	NM_003400.3	c.1711G>A	p.E571K	EX15	1.3%
*CDKN2A*	NM_000077.4	missing	-	9p21.3	0.5
*CDKN2B*	NM_004936.3	missing	-	9p21.3	0.4

1. The table only lists the functional variations in the coding regions of important genes; 2. Mutation frequency refers to the proportion of mutations found at this locus in the total of wild type and mutant alleles during allele testing; 3. The normal value of copy number is 1.

"*" is used for nucleotide numbering and indicates the translation termination codon.

"-" means blank.

## Discussion

Initially, we considered the sudden loss of vision in his right eye as pituitary apoplexy, but we found that the tumor had invaded the right optic canal and did not find any evidence of pituitary apoplexy during the surgery. Reviewing the diagnosis and treatment process of this case, his medical history, clinical symptoms, signs, laboratory findings and radiological characteristics were consistent with pituitary adenoma, so it was easy to be misdiagnosed as pituitary apoplexy. A retrospective study showed that the clinical manifestations of pituitary lymphoma were similar to those of invasive pituitary adenoma, and half of patients show clinical and/or laboratory evidence of hypopituitarism at diagnosis. In addition, 50% of the patients had visual field defects and 40% had symptoms of cranial nerve involvement (5). Clinical studies have shown that most B-LBL patients present with lower stage lesions, mainly consisting of osteolytic bone lesions (26%) and skin lesions (23%), and rare findings included mediastinal or pleural disease (11%), isolated bone marrow disease (13%), isolated lymph node (13%), or visceral disease (4%). Only a minority of patients (6%) have central nervous system manifestations (6). Meyer et al. used molecular inversion probe technology to analyze the genetic data of 23 B-LBL patients, and found that *CDKN2A/B* deletions were the most common alteration identified in 6/23 (26%) B-LBL cases. IKZF1 and *PAX5* deletions were observed in 13% and 17% of B-LBL (7). *TP53* is the most frequently mutated gene in human cancer, and is closely related to the occurrence and development of tumors (8). Mutations in the *TP53* gene detected in multiple NHL patients, and the detection frequencies in diffuse large B-cell lymphoma (DLBCL), transformed follicular lymphoma (tFL) and Burkitt lymphoma (BL) patients were 21%-31.7% (9–11), 29%-80% (12) and 33%(12), respectively. Mutations in the TP53 gene are extremely rare in patients with B-LBL (13).

**Figure 2:**
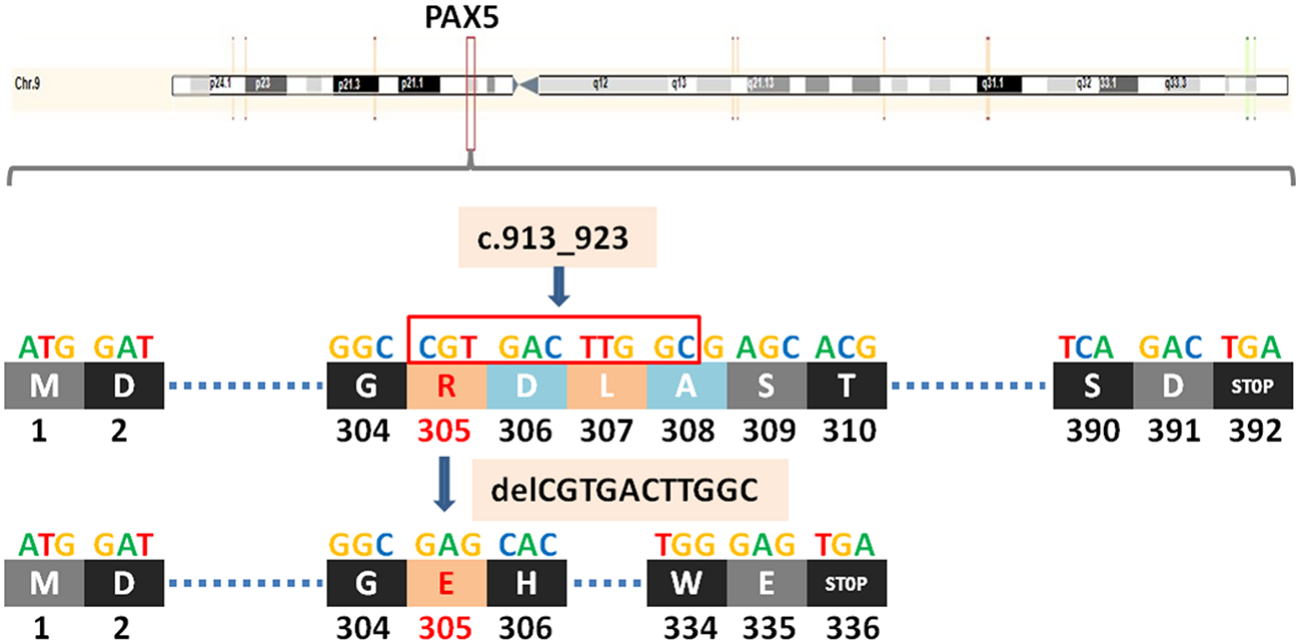
A newly discovered frameshift mutation in PAX5, which leads to a substitution of the arginine at position 305 to glutamic and termination at position 336. ATG (base sequence) transcription translates to methionine (M,start codon); GAT/GAC transcription translates to aspartic acid (D); GGC transcription translates to glycine (G); CGT transcription translates to arginine (R); GAG transcription translates to glutamic acid (E); CAC transcription translates to histidine (H); TTG transcription translates to leucine (L); GCG transcription translates to alanine (A); AGC/TCA transcription translates to serine (S); ACG transcription translates to threonine (T); TCA transcription translates to serine (S); TGG transcription translates to tryptophane (W); TGA transcription translates to stop codon.

*PAX5* is a member of the PAX transcription factor family. The main feature of this gene family is the highly conserved DNA binding motif paired box. *PAX5* plays an important role in B cell differentiation, and participates in the regulation of B lymphocyte specific target gene - CD19 gene. B cell-specific activator protein (BSAP) encoded by Pax5 gene plays a decisive role in the early directed differentiation of B cells. In the process of hepatocarcinogenesis, *PAX5* inhibits hepatocarcinogenesis by inhibiting cell proliferation and regulating p53 signaling pathway to induce apoptosis. It can be used as an auxiliary marker in diagnosing classical Hodgkin lymphoma and B-NHL (14, 15). Studies have shown that in B lymphoma, *PAX5* promotes lymphomagenesis through stimulation of B cell receptor signaling (16). However, other studies have shown that *PAX5* silencing promotes mantle cell lymphoma (MCL) cell proliferation and that *PAX5* overexpression induces MCL cell death (17). Mutations in the *PAX5* gene are detected at a frequency of 7% in patients with B-cell progenitor acute lymphoblastic leukemia (BCP-ALL) (18). *PAX5* c.913_923delCGTGACTTGGC (p.R305Efs*32) is a frameshift mutation, which leads to a substitution of the arginine at position 305 to glutamic and termination at position 336 and causes the transcription to end prematurely (**Figure 2**), resulting in functionally impaired proteins, or loss of protein expression through nonsense-mediated mRNA degradation (NMD).

**Figure 3:**
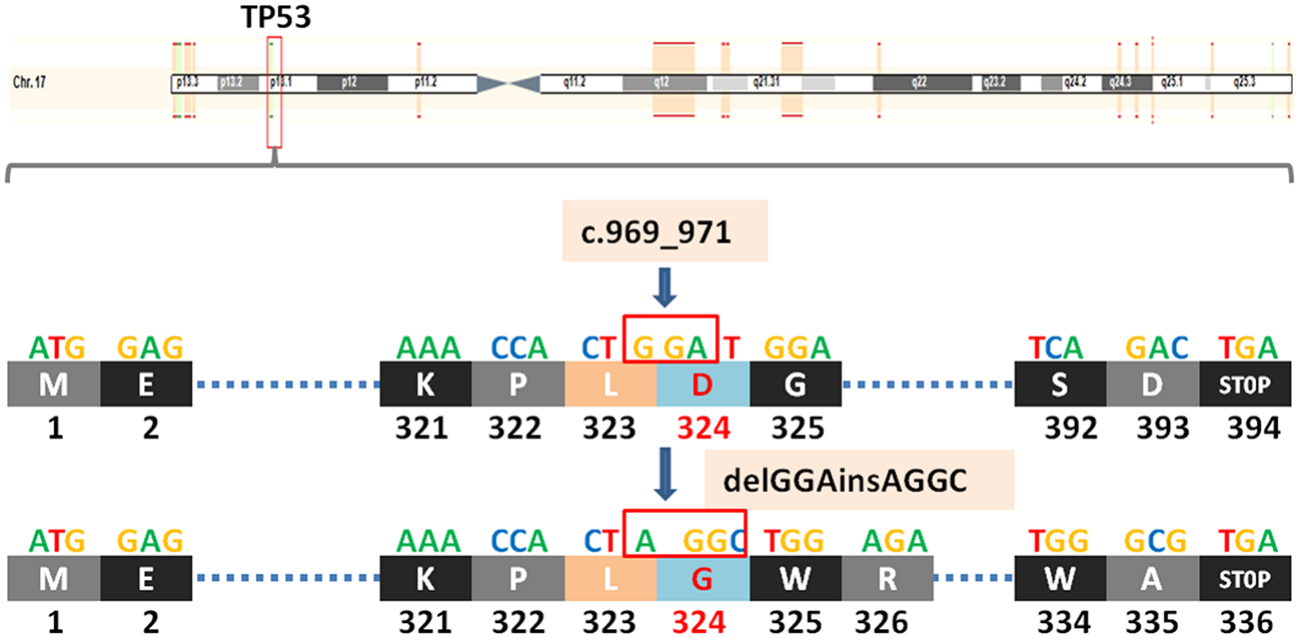
A newly discovered frameshift mutation in TP53, which leads to a substitution of the aspartic at position 324 to glycine and termination at position 336. ATG (base sequence) transcription translates to methionine (M,start codon); GAG transcription translates to glutamic acid (E); AAA transcription translates to lysine (K); CCA transcription translates to proline (P); CTG/CTA transcription translates to leucine (L); GAT/GAC transcription translates to aspartic acid (D); GGA/GGC transcription translates to glycine (G); TCA transcription translates to serine (S); TGG transcription translates to tryptophane (W); AGA transcription translates to arginine (R); GCG transcription translates to alanine (A); TGA transcription translates to stop codon.

*TP53* (tumor protein P53) is a tumor suppressor gene. The protein it encodes, P53, is a DNA binding protein involved in a variety of biological processes. P53 protein regulates target gene expression in response to various cellular stresses, thereby inducing cell cycle arrest, cell apoptosis and senescence, DNA repair, or changes in metabolism. In the cell cycle, when the DNA in the cell is damaged or defective, the P53 protein will arrest the cell cycle in G1 and G2 phases, and start the corresponding repair mechanism to repair the damaged or defective DNA. If the repair fails, the P53 protein initiates the apoptosis mechanism to clear the damaged cells, thus achieving the regulatory function. *TP53* c.969_971delGGAinsAGGC (p.D324Gfs*13) is also a frameshift mutation, which leads to a substitution of the aspartic at position 324 to glycine and termination at position 336 and causes the transcription to end prematurely(**Figure 3**), resulting in functionally impaired proteins, or loss of protein expression through nonsense-mediated mRNA degradation (NMD).

*PAX5* c.77T>G (p.V26G) is a missense mutation, which leads to a substitution of the valine at position 26 to glycine. SIFT and PolyPhen-2 provide an in silico prediction of the functional consequences of missense mutations. The former appears to be harmless, while the latter is on the opposite side. *XPO1* is a proto-oncogene and Involved in the regulation of mitosis. *XPO1* c.1711G>A (p.E571K) is a missense mutation, which leads to a substitution of the glutamic at position 571 to lysine. It can be used as a novel biomarker for classical Hodgkin lymphoma and helps to capture the pathogenesis of classical Hodgkin lymphoma (19). *CDKN2A* and *CDKN2B* are tumor suppressor genes. Loss of *CDKN2A* may lead to the loss of p16ink4a which is a CDK4/6 inhibitor. In turn, CDK4/6 will be activated, resulting in dysregulation of cell proliferation (20, 21). *CDKN2B* copy number variation is the result of deletion of the *CDKN2A-CDKN2B* locus.

## Conclusion

Sellar lymphoma has no special imaging features, which presents a huge challenge to both accurate diagnosis and personalized therapy. Although sellar lymphoma is extremely rare, it should always be considered in the diagnosis of sellar tumors, and early diagnosis and systematic treatment are the key to improve the prognosis of B-LBL patients.

## Data availability statement

The datasets presented in this study can be found in online repositories. The names of the repository/repositories and accession number(s) can be found in the article/supplementary material.

## Ethics statement

Written informed consent was obtained for the publication of this case report.

## Author contributions

XH, YW, DW and FB were involved in the surgery. PL, XX and CW did literature search. YW, YY and QW drafted the paper. XH revised the paper. All authors contributed to the article and approved the submitted version.
